# A diabetic patient presenting with stiff hand following fasciectomy for Dupuytren's contracture: A case report

**DOI:** 10.1186/1757-1626-1-277

**Published:** 2008-10-27

**Authors:** Katia Fournier, Nikolaos Papanas, Jonathan P Compson, Efstratios Maltezos

**Affiliations:** 1 Hand Therapy Department, King's College Hospital, London, SE5 9RS, UK; 2 Outpatient Clinic of Obesity, Diabetes and Metabolism, Second Department of Internal Medicine, Democritus University of Thrace, Alexandroupolis, 68100, Greece; 3 Orthopaedic Department, Upper Limb Unit, King's College Hospital, London, SE5 9RS, UK

## Abstract

Reported is the case of a 68-year-old male presenting with severe wrist and hand stiffness following surgery for a Dupuytren's contracture. Complications of surgery or rehabilitation and complex regional pain syndrome were excluded as factors explaining this stiffness. Given the patient's diabetes mellitus and the striking similarity with the typical diabetic stiff hand, it is suggested that diabetes may have contributed to the development of the complication.

## Introduction

Dupuytren's disease is a fibroproliferative disorder of unknown origin causing palmar nodules and flexion contracture of the digits [1]. The treatment of choice is surgical excision of the affected palmar fascia. "Flare reaction", is a complication occurring in 5 to 25% of patients undergoing surgery [2,3]. It usually occurs during the third or fourth week post surgery and is characterised by oedema, redness, increased pain and stiffness in patients who show good progress during the first few weeks post-operatively [4].

## Case presentation

A 68-year-old right-hand dominant Caucasian male developed severe wrist and hand stiffness following a selective fasciectomy of the right middle and little fingers for Dupuytren's contracture. The patient described full finger flexion prior to surgery. The contracture had been present for approximately 5 years. There was no presence of ectopic lesions, no significant history of Dupuytren's disease in the family and no alcohol consumption or smoking. Co-morbidities included high blood pressure and type 2 diabetes mellitus of 10 years' duration. Diabetes control was average (HbA1c = 7.2%), but the patient reported occasional very high glucose readings above 13.9 mmol/l (250 mg/dl) during the past three years. Full blood count was normal, as were liver function tests, urea and creatinine. Urine examination was negative for proteinuria. The medication taken was amlodipine 10 mg od, bendroflumethiazide, 2.5 mg od, gliclazide 80 mg bd, metformin 500 mg bd and simvastatin 20 mg od. None of his medication has been shown to induce fibrosis or stiffness.

**Figure 1:**
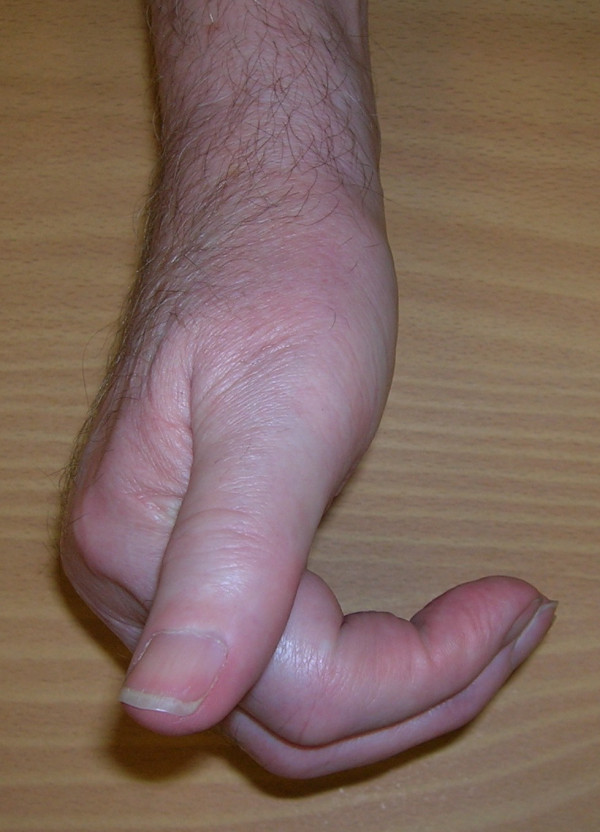
Composite finger flexion at 6 months post-operatively.

**Figure 2:**
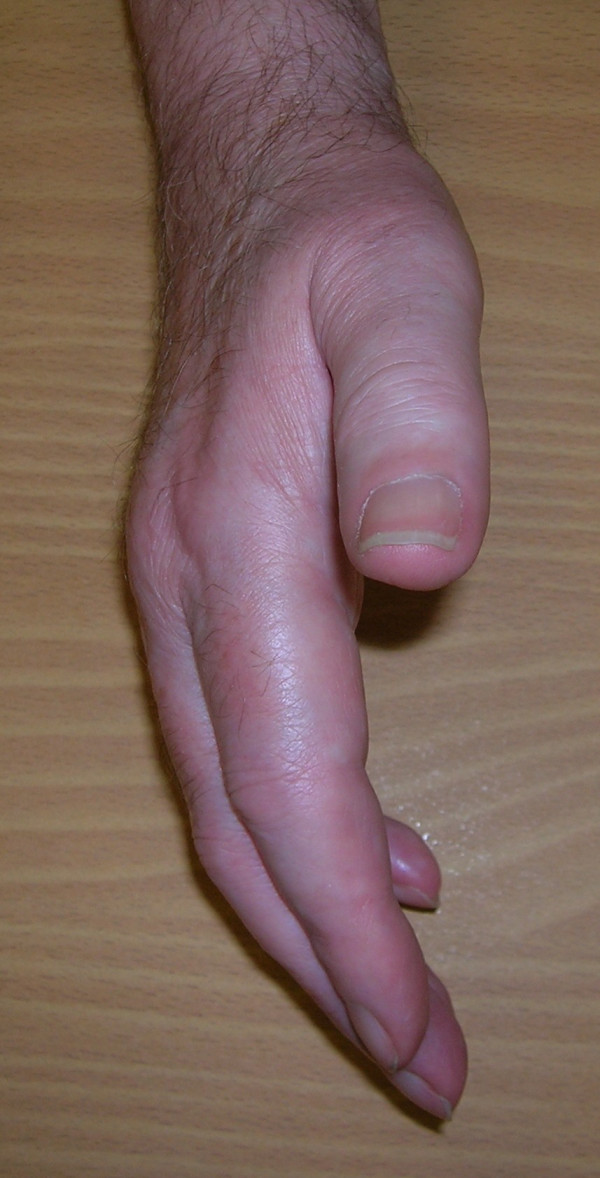
**Finger extension at 6 months post-operatively.** The hand posture is similar to the typical diabetic stiff hand.

Surgery was performed by an experienced upper limb surgeon under general anaesthesia, using standard incisions (Bruner type). The neurovascular bundles were identified and preserved. Full extension of both fingers was achieved during surgery. A plaster palmar slab with bulky dressing was applied and the patient was reviewed by the surgeon after a week. No signs of complications such as infection, wound dehiscence, haematoma or complex regional pain syndrome were identified. Hand therapy was initiated at that visit by an experienced therapist (first author) using the centre's usual protocol. During rehabilitation, the patient reported minimal pain, no symptoms of nerve damage, and good compliance with his home exercise programme. The wound was nearly fully healed at 2 weeks except for two small areas that took 4 weeks to heal, which is quite usual following this type of surgery. Active range of motion measured with a goniometer and grip strength measured with a Jamar dynamometer is presented in Table 1. Finger flexion was limited as expected at week 2, but got slightly worse at week 3. Of interest, the increase in stiffness was associated with the development of pitting oedema of the hand. Range of motion and oedema were monitored every week and, apart from a gradual loss of finger extension, showed little changes in the first 12 weeks. Oedema and stiffness did not respond to the usual treatment modalities. While oedema had completely resolved at 9 months, the range of motion and strength remained severely limited. Figures 1 and 2 illustrate the patient's finger extension and flexion 6 months post-operatively.

**Table 1:** Active range of motion of the wrist and fingers and grip strength at 2 weeks, 3 weeks, 12 weeks and 9 months post-operatively.

		2 weeks	3 weeks	12 weeks	9 months (discharge)
Wrist		Not available	Not available	50/50	65/55

Index	MCPJ	0/60	0/50	-10/60	0/80
	
	PIPJ	0/75	0/70	0/60	0/85
	
	DIPJ	0/30	0/30	0/30	0/50

Middle	MCPJ	-10/55	-20/55	-25/55	-15/80
	
	PIPJ	0/60	0/55	-20/55	-20/80
	
	DIPJ	0/25	0/25	0/30	0/45

Ring	MCPJ	0/45	-20/50	-5/55	0/75
	
	PIPJ	0/60	0/55	0/55	0/85
	
	DIPJ	0/25	0/30	0/20	0/25

Little	MCPJ	-10/30	0/40	0/50	0/55
	
	PIPJ	0/50	0/45	-20/40	-50/85
	
	DIPJ	0/25	0/30	0/25	0/40

Grip strength		Not available	Not available	3 Kg	13 Kg

Reported are degrees of extension/flexion, a negative value being a lack of extension. MCP: metacarpophalangeal joint, PIPJ: proximal interphalangeal joint, DIPJ: distal interphalangeal joint, Kg: kilograms.

## Discussion

The patient described appears to have developed a severe "flare reaction". Importantly, this reaction could not be attributed to surgical complications, as no such complications were noted during the operation itself or in the early post-operative phase. It is also unlikely that the rehabilitation protocol triggered hand stiffness, since we employed an adapted version of a protocol minimising stiffness [5], which has been routinely used in our centre during the past 4 years without complications. Finally, complex regional pain syndrome was also excluded, because the patient reported minimal pain.

Given that no obvious causes for the patient's flare reaction were found, it is worth considering the potential role of diabetes. Indeed, the hand posture was very similar to the typical diabetic stiff hand, also called limited joint mobility (LJM) as shown in Figure 2[6,7]. Thus, the patient initially presented with one of the traditional musculoskeletal disorders of diabetes (Dupuytren's contracture) and, eventually, developed another traditional diabetic musculoskeletal disorder (LJM) which are both attributable to his long-term hyperglycaemia [7,8]. It is conceivable (though not proven) that diabetes may be an underlying factor of both complications, the latter having been triggered by surgery.

## Conclusion

"Flare reaction" is a significant complication of Dupuytren's surgery. To the best of our knowledge, there seems to be no investigation into the potential causes and no detailed description of the severity of this complication. Diabetes mellitus may represent a hitherto unrecognised factor, and its role clearly deserves further investigation.

## Consent

Written informed consent was obtained from the patient for publication of this case report and accompanying images. A copy of the written consent is available for review by the Editor-in-Chief of this journal.

## Competing interests

The authors declare that they have no competing interests.

## Authors' contributions

KF analysed and interpreted the patient data regarding the hand stiffness, NP and EM analysed and interpreted the patient data regarding diabetes and JC analyzed the surgical data. All authors read and approved the final manuscript.
